# Sebaceous Carcinoma of the Breast: A Case Report and Review of the Literature

**DOI:** 10.7759/cureus.89443

**Published:** 2025-08-05

**Authors:** Fadwa Haboub, Othmane Zouiten, Mohamed El Fadli, Leila Afani, Rhizlane Belbaraka

**Affiliations:** 1 Medical Oncology, Mohammed VI University Hospital of Marrakech, Marrakech, MAR; 2 Medical Oncology, Faculty of Medicine and Pharmacy of Marrakech, Cadi Ayyad University, Marrakech, MAR; 3 Oncology, Mohammed VI University Hospital of Marrakech, Marrakech, MAR; 4 Medical Oncology, Mohammed VI University Hospital of Marrakech, Marrakesh, MAR

**Keywords:** chemoresistance, immunohistochemistr, metastatic, primary breast neoplasm, sebaceous carcinoma of the breast

## Abstract

Sebaceous carcinoma of the breast is a rare and poorly understood variant of metaplastic breast carcinoma. Its histogenesis, clinical behavior, and optimal management remain unclear due to the limited number of reported cases.

We report the case of a 78-year-old woman presenting with a six-month history of a right axillary mass and inflammatory changes in the right breast. Clinical and radiologic assessments indicated an advanced-stage lesion. Histological analysis from a core needle biopsy revealed solid nests and tubules with sebaceous differentiation. Immunohistochemistry confirmed a triple-negative profile, with high proliferative activity. The patient received neoadjuvant chemotherapy without clinical improvement. Disease progression was noted despite a switch to a platinum-based regimen, followed by further deterioration after additional treatment. The patient was eventually lost to follow-up.

This case highlights the aggressive nature and treatment resistance of this carcinoma and underscores the need for further investigation to better define its characteristics and guide therapeutic strategies.

## Introduction

Sebaceous carcinoma (SC) of the breast is a rare type of primary breast carcinoma, accounting for 0.1-2% of invasive breast carcinomas [1]. Because of its rarity and controversial histogenesis and pathophysiology, the SC of the breast is difficult to elucidate [2]. Few cases have been reported in the literature to date. We report a new case of this rare entity, taking into account its specific characteristics through a review of the literature.

## Case presentation

**Figure 1:**
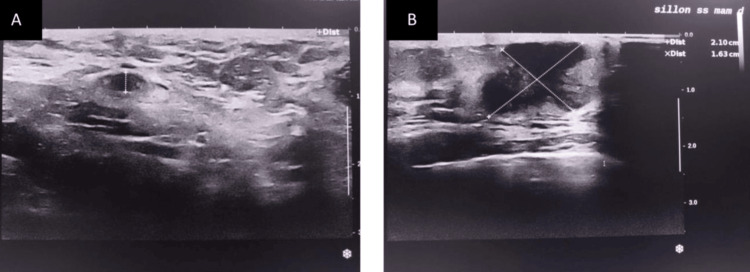
Ultrasound mammography showing bilateral mastoid plaques, a suspicious right retro-nipple lesion, and largely necrotic ipsilateral lymphadenopathy. Classified as ACR BIRADS 5 on the right and BIRADS 3 on the left.

A 78-year-old woman with a history of hypertension presented with a 6-month history of a right axillary mass identified through self-examination. She was otherwise in good general health, with no fever or systemic symptoms. On clinical examination, a 7 cm nodular mass was noted in the right axillary extension. The mass was fixed to both the skin and underlying planes and appeared inflamed. The right breast showed signs of local inflammation, while the left breast and other lymph node areas were unremarkable. According to the TNM classification (8th edition), the case was clinically staged as cT4d N2a Mx. Ultrasound and mammography revealed bilateral mastoid plaques, a suspicious lesion in the retroareolar region of the right breast, and largely necrotic homolateral axillary lymphadenopathy. The imaging findings were categorized as ACR BI-RADS 5 on the right and BI-RADS 3 on the left (Figure 1).

**Figure 2:**
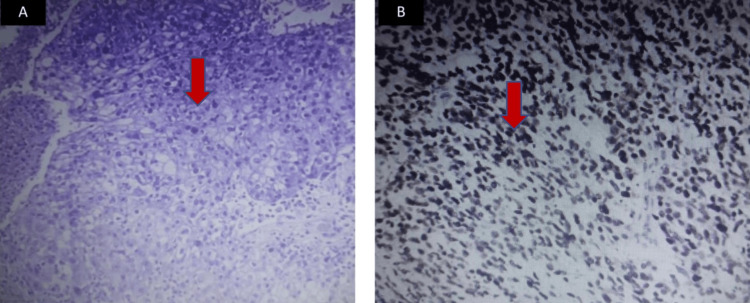
Histological and immunohistochemical features of sebaceous carcinoma of the breast showing a cystic 4 x 2 cm lesion with poorly demarcated epidermis, solid masses with cellular tubules around keratin pearls, clear vacuolar cytoplasm, and sebaceous differentiation. Immunohistochemistry positive for EMA, GATA3, CK7; negative for P63, hormone receptors, mammaglobin, HER2 (+1); high Ki-67 index (90%)

A surgical biopsy of the right breast nodule was performed. Histological examination revealed a 4 × 2 cm cystic structure, poorly circumscribed, lined by epidermis and composed of solid nests and tubular structures centered around keratin pearls. Focal areas of sebaceous differentiation were noted, characterized by cells with abundant, clear vacuolated cytoplasm. These histological features were consistent with sebaceous carcinoma (Figure 2). Immunohistochemistry showed positivity for epithelial membrane antigen (EMA), GATA3, and cytokeratin 7 (CK7), while p63, estrogen receptor (ER), progesterone receptor (PR), mammaglobin, and HER2 (score +1) were negative. The Ki-67 proliferation index was high (90%). The tumor was thus classified as a triple-negative sebaceous carcinoma of the breast.

A staging work-up (TAP CT scan) showed no evidence of distant metastases at baseline. The patient received neoadjuvant chemotherapy following a sequential regimen: 4 cycles of doxorubicin (60 mg/m²) and cyclophosphamide (600 mg/m²) every three weeks (AC60), followed by 12 weekly doses of paclitaxel (80 mg/m²). Disease progression was observed during treatment, with no clinical response and the emergence of a skin permeation nodule after 4 AC cycles and the full paclitaxel course. A platinum-based regimen was then initiated, combining weekly paclitaxel (80 mg/m²) with carboplatin (AUC 2).

**Figure 3:**
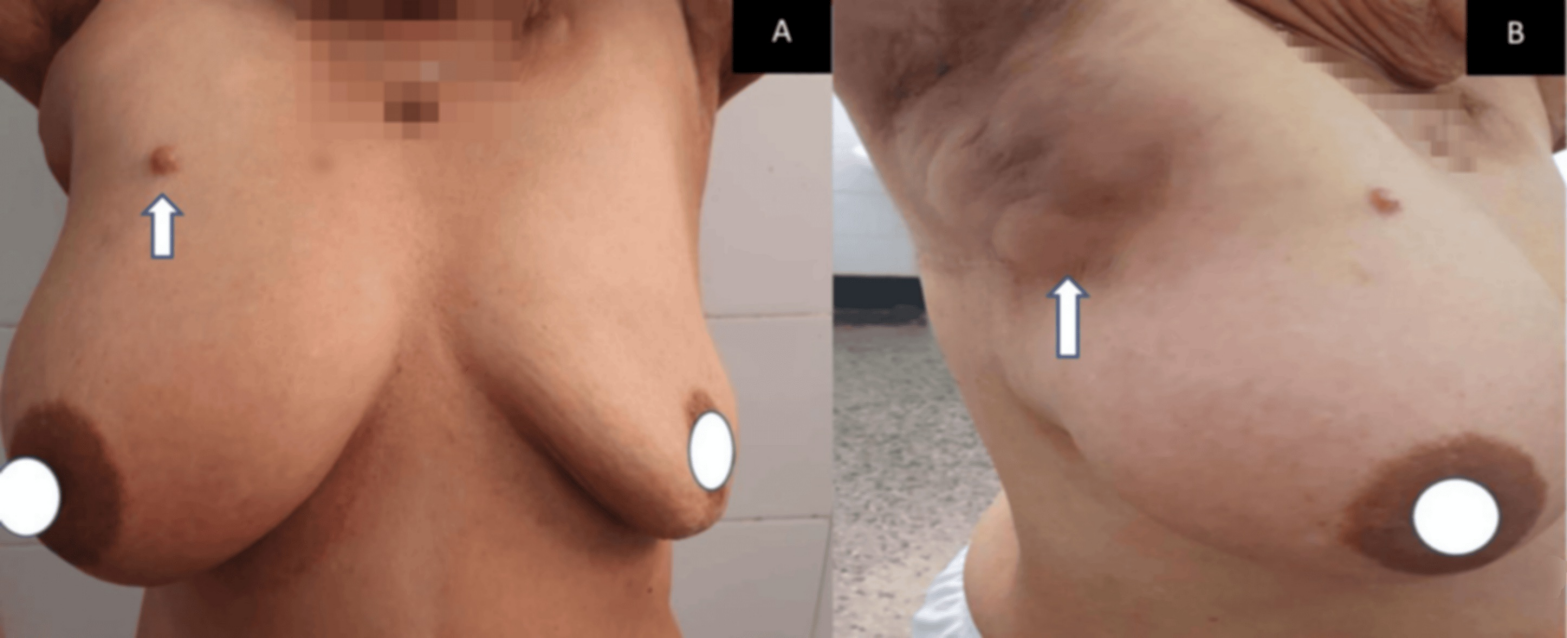
Clinical photograph showing multiple permeation nodules on the right breast extending to the chest wall, with fistulation of one nodule and skin ulceration overlying enlarged right axillary lymphadenopathy.

**Figure 4:**
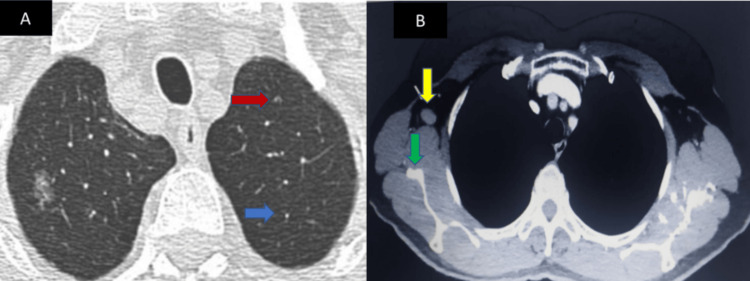
Axial CT scan showing four significant findings highlighted with color-coded arrows Red arrow: significant enlargement of the necrotic mass in the right axillary region; blue arrow: two micronodules in the middle lobe; green arrow: a left basal pulmonary nodule; yellow arrow: mediastinal millimetric lymph node

After three cycles, clinical reassessment showed further disease progression: multiple new skin permeation nodules involving the chest wall, fistulation of one lesion, increased size of the right axillary adenopathy with overlying skin ulceration (Figure 3), grade 3 peripheral neuropathy, and elevated CA 15-3 levels (84.5 U/mL, previously 23.6 U/mL). A follow-up TAP CT scan demonstrated marked enlargement of the necrotic mass in the right axillary region, the appearance of new pulmonary micronodules in the middle lobe, a posterior basal nodule in the left lung, mediastinal lymphadenopathy, and a suspicious hepatic lesion in segment III, suggestive of metastatic spread (Figure 4). The patient was then switched to vinorelbine-based chemotherapy and received symptomatic treatment for peripheral neuropathy. Unfortunately, she was subsequently lost to follow-up. 

## Discussion

Sebaceous carcinoma of the breast is an exceedingly rare entity, accounting for approximately 0.1-2% of all breast carcinomas [1]. It belongs to the heterogeneous group of metaplastic breast carcinomas. According to the World Health Organization (WHO), sebaceous carcinomas are classified as infiltrating ductal carcinomas exhibiting partial or complete metaplastic transformation. When metaplasia is complete, the tumor may present as a pure squamous cell carcinoma. Some authors have also proposed a myoepithelial origin for these tumors [2].

Epidemiologically, the reported age at diagnosis ranges from 30-83 years, with a peak incidence around 55 years [3], which is similar to that observed in other types of breast carcinoma. To date, no definitive predisposing factors have been identified.

Clinically, sebaceous carcinomas often present as palpable masses with an average size of 5 cm (range: 1-10 cm), and may occasionally be associated with abscess formation or skin ulceration. These features can raise concerns regarding a possible secondary origin or cutaneous involvement [4]. Radiologically, no pathognomonic imaging features have been described. However, ultrasound typically reveals a round, non-spiculated, partially irregular mass with a necrotic or cystic center, mimicking the appearance of an abscess or pseudocyst [4,5].

The diagnosis is established histologically, either by core needle biopsy or fine-needle aspiration, as was done in our case [6]. Histological examination should assess for the presence of an adenoid component and exclude a local extension of cutaneous squamous cell carcinoma or metastasis from an extra-mammary primary tumor. Therefore, the diagnosis of primary sebaceous carcinoma of the breast is made only after ruling out secondary origins, including skin or nipple involvement and distant metastatic disease.

Immunohistochemically, sebaceous carcinomas express high molecular weight cytokeratins such as CK14, CK6, and CK17 [5]. Hormone receptors (estrogen and progesterone) and HER2 are typically negative, which classifies most of these tumors as triple-negative. A high proliferative index, reflected by an elevated Ki-67 score, is commonly observed [7], as was the case in our patient.

The therapeutic approach generally follows the same principles as for infiltrating ductal carcinomas of similar size, stage, and molecular subtype [8]. Some authors, such as Dejager et al. [9], have reported the use of neoadjuvant chemotherapy regimens containing platinum salts and 5-fluorouracil to reduce tumor burden prior to surgery. The prognosis of sebaceous carcinomas remains controversial. While some studies suggest an outcome similar to that of conventional ductal carcinomas when matched by stage [10], others report a more aggressive clinical course. Key prognostic factors include tumor size, axillary lymph node involvement, presence of necrosis, and cellular acantholysis [10]. Overall, the five-year survival rate is estimated at approximately 50% [11].

In recent years, molecular targets such as the epidermal growth factor receptor (EGFR) have emerged as potential therapeutic avenues, especially in triple-negative and metaplastic subtypes, offering new hope for improving clinical outcomes [11].

## Conclusions

Sebaceous carcinoma of the breast is a rare metaplastic breast tumor with clinical and radiological features identical to those of ductal carcinoma. Its prognosis appears to be guarded, hence the need for further studies to provide a better histopathogenic approach in order to develop specific management.
